# The economic cost of malaria in Brazil from the perspective of the public health system

**DOI:** 10.1371/journal.pgph.0003783

**Published:** 2024-10-18

**Authors:** Mônica V. Andrade, Kenya Noronha, Valéria Silva, Henrique Bracarense, Lucas Carvalho, Daniel Nogueira da Silva, Aline Souza, André Soares Motta-Santos, Cassio Peterka, Marcia C. Castro

**Affiliations:** 1 Center of Regional Planning and Development, Universidade Federal de Minas Gerais (Cedeplar-UFMG), Belo Horizonte, Minas Gerais, Brazil; 2 Secretaria de Vigilância em Saúde e Ambiente, Ministério da Saúde, Brasília, DF, Brazil; 3 Department of Global Health and Population, Harvard T.H. Chan School of Public Health, Boston, Massachusetts, United States of America; Soongsil University, KOREA, REPUBLIC OF

## Abstract

This paper presents an economic evaluation aimed at estimating malaria-related health care expenditures in Brazil from the perspective of the public health system. Comprehensive estimates of the costs of malaria remain scarce, in part because of limited data. Brazil has a universal health system (Sistema Único de Saúde-SUS) in which all cases of malaria are diagnosed and treated. In addition, antimalarial drugs are only available through the SUS. We compiled comprehensive data from multiple administrative sources. Expenditure indicators were calculated for municipalities and states in the Brazilian Amazon, a region where more than 99% of the country’s malaria cases are concentrated. We also developed a digital platform that allows interactive visualization of the malaria cost indicators, disaggregated by cost type and geography. Our results show that control and prevention activities are the primary cost in all states, followed by human resources and disease treatment. Expenditures per malaria notification in the Amazon ranged from PPP (purchasing power parity)-US$59.00 (2017) to PPP-US$77.00 (2016), while per capita expenditures remained relatively stable at around PPP-US$3.50. The malaria cost estimates presented here contribute to a better negotiation of the financial resources needed by the NMCP and are one of the critical pieces of information for a strategic plan of activities needed to achieve the elimination goal.

## Introduction

Malaria is caused by *Plasmodium* parasites, which are transmitted to humans through the bite of infected *Anopheles* mosquitoes. In 2021, an estimated 274 million malaria cases and 619 thousand malaria deaths were reported, with the vast majority concentrated on the African continent [1]. Still concentrated in low-income areas, malaria contributes to deep poverty [2]. The main drivers are the impact on work and school absenteeism associated with its symptoms, which include fever, cough, aches, chills, nausea, and vomiting. Typically, individuals may experience multiple malaria reinfections during their lifetime, resulting in lost productivity and earnings. In 2019, the burden of malaria, as measured by disability-adjusted life years (DALYs), ranked 19^th^ among all causes and 4^th^ when considering only infectious diseases [3].

Empirical evidence has shown that countries with high malaria transmission rates have experienced significant reductions in annual economic growth [4, 5]. In addition, government and out of pocket expenditures on malaria have increased worldwide over the past decade [6, 7]. Estimates of malaria expenditure at a broad level are valuable because they fill a major gap in our knowledge of malaria financing. Economic costs are often devided into two categories: direct (treatment and control) and indirect (value of time lost due to morbidity or premature mortality). These costs are borne by the health care system, individuals, their households, and the broader community.

Studies of the economic costs of malaria remain scarce worldwide [8, 9]. Existing evidence focuses primarily on African countries, especially those with a high malaria burden, and reveals a research gap for Latin American countries [9–12]. Few studies allow for comparability across countries. Devine et al. [13] estimated direct and indirect household costs in a multicenter study, and found that the average total cost varied from US$8.7 in Afghanistan to US$254.7 in Colombia. Haakenstad et al. [6] estimated the global spending on prevention and treatment of malaria based on the System of Health Accounts of 106 countries. The figures reached US$4.3 billion (95% UI 4.2–4.4) in 2016, including both government and out-of-pocket spending.

Malaria costing studies at the country or regional level often differ in several respects: (i) types of costs considered, (ii) number of years of data used, (iii) choice of cost analysis perspective (provider or societal), (iv) geographic coverage, and (v) parasite species considered. These differences are a major obstacle to comparing results across studies and suggest the lack of a standardized conceptual framework for estimating malaria-related expenditures. In the context of malaria, most studies focus on the household perspective because families bear substantial indirect costs. Estimating costs from a health system perspective is often challenging due to the lack of information systems that facilitate the tracking of disease-specific expenditures [9]. Jowett and Miller [14] conducted a comprehensive estimate of malaria treatment and control expenditures in Tanzania, considering private, government, and international donors expenditures, and found that the economic burden of malaria was 1.1% of the GDP, of which 20% was attributed to the government, 71% to families, and 9% to donors. These results provide important empirical evidence on the challenges that need to be overcome to achiee comprehensive interventions with national coverage. One of the most challenging tasks is to define apportionament assumptions to obtain specific numbers for each disease.

Here we present a costing study aimed at estimating malaria-related health care expenditures in Brazil from the perspective of the public health system. Expenditure indicators were estimated for municipalities and states in the Brazilian Amazon, a region where more than 99% of the country’s malaria cases are concentrated. Brazil was chosen for four reasons. First, the country has a universal health system (called SUS); all malaria cases are diagnosed and treated in the public system, and antimalarial drugs are only available through the SUS (they are not sold in drugstores). Second, Brazil routinely collects surveillance and accounting information. Third, there are some empirical studies that estimate the cost of malaria in Brazil. However, none of them has provided a national representation or a comprehensive estimate of the economic costs of malaria in the Brazilian Amazon. One study has calculated the costs of malaria in a low-endemic area of Brazil, the city of Manaus, and it was focused on pregnant women receiving care at a single health facility [15]. Other studies have focused on specific aspects, such as the cost of G6PD deficiency in male patients with Plasmodium vivax malaria treated with primaquine [16], the cost-effectiveness analysis of a rapid diagnostic test to detect the G6PD deficiency [17], and the cost-effectiveness analysis of malaria diagnostics [18, 19]. Fourth, Brazil has set a goal to eliminate malaria by 2035, and cost estimates are important to support this goal. Based on our findings, we have also developed a user-friendly and interactive platform that provides malaria expenditures, disaggregated by state and municipal levels and by cost components.

## Materials and methods

### Conceptual framework

To define the cost components of malaria, we adapted the conceptual framework developed by Castro, Wilson, and Bloom [20], which categorizes the economic burden of dengue into four domains (illness, surveillance and reporting, control and prevention, and outbreak management) and three stakeholders who bear the costs (health care providers, individuals or households, and communities). Given the availability of data on malaria, we categorized public expenditures into three domains: (i) illness, including all costs associated with malaria diagnosis and treatment; (ii) surveillance and control, combining the costs of epidemiologic surveillance, insecticide and long-lasting insecticide-treated net (LLIN) procurement, and screening of donated blood bags; and (iii) human resources, including the salaries of microscopists and professionals involved in routine surveillance and control activities (disease agents and community health workers), as well as government financial incentives for microscopists (S1 Fig).

### Data

We collected information from multiple data sources (described in S1 Table) for all municipalities in the Brazilian Amazon for the years 2015 to 2019. The Brazilian Amazon comprises nine states: Acre, Amapá, Amazonas, Maranhão, Mato Grosso, Pará, Rondônia, Roraima and Tocantins. We chose a five-year period to account for possible seasonal variations that may arise due to changes in malaria incidence or administrative/policy decisions. We also did not include data after 2020 to avoid bias due to the COVID-19 pandemic. All expenditure data were obtained at current prices and converted to purchasing power parity (PPP, 2020 US$) using the Campbell and Cochrane Economics Methods Group (CCEMG)–Evidence for Policy and Practice Information and Coordinating Center (EPPI-Centre) cost converter [21].

### Cost calculation

Table 1 summarizes the calculation method and assumptions for each cost component detailed by the domains described in the conceptual framework. Some cost components were quantified directly from information available in administrative records, while others required specific assumptions. First, because malaria cases are often diagnosed and reported in small health units or laboratories, and involve only testing and drug dispensing, we assumed that only severe malaria cases require outpatient consultation. Here, severe malaria follows the definition of the Ministry of Health: sheme 11 of the Sivep [22]. Second, for treatment, the cost of drugs was estimated for each regimen stratified by age group, taking into account government purchases. Third, since all data on insecticide and LLIN procurement are available only for federal units, we considered the total population of each municipality to allocate the numbers. Finally, since each suspected case reported in SIVEP-Malaria includes the ID code of the endemic disease control agent, we considered the number of unique codes reported each year as the total number of agents working on the program. These agents are involved in malaria surveillance activities and also manage health services in health units. As a result, human resources expenditures include the costs associated with all malaria patients, both severe and non-severe.

**Table 1:** Calculation and assumptions for each cost component by domain.

Domain	Cost componente	Calculation method	Notes
Illness	Diagnosis test	# of malaria notifications * unit value of the diagnosis test	Cure verification slides were included in the total # of notifications. Calculations were disaggregated by type of test: thick blood smear and rapid test.
	Treatment (medical consultation)	# of severe malaria notifications * value of specialized consultation	We assumed that only patients with severe malaria received a consultation. Severe malaria follows the Ministry of Health’s definition [22]
	Treatment (hospitalization)	Total expenses with all hospital procedures for the treatment of malaria, independently of ICD code; and hospital admissions with ICD codes B50, B54, P37.3, or P137.4.	
	Treatment (medication)	# of confirmed malaria cases by parasite and age of the patient * value of each treatment regimen	Medication costs were estimated considering 12 possible first-line therapeutic regimens defined in the Practical Guide [22]. In order to estimate the cost of each regimen, all purchases made by the Ministry of Health were taken into account. The ultimate unit value was calculated as a weighted mean, with weights assigned based on the quantity of each purchase. The final cost with treatment was calculated by assigning a regimen type to each case based on parasite specie and age of the patient, and then applying the cost of that regimen.
Surveillance and Control	Insecticides and long-lasting insecticide-treated nets (LLINs)	Estimated expenses with insecticides for indoor residual spraying (IRS), truck spraying, and LLINs	Since data are only available for each Federal Unit, expenses were apportioned to municipalities based on population size
	Screening blood donation bags	# of blood bags * value of the thick blood smear test	
	Surveillance	Estimated expenses with malaria surveillance	Surveillance costs are not discriminated by disease. We assumed that expenses with malaria surveillance are proportional to the relative contribution of malaria cases among all cases of diseases of mandatory notification. To obtain cost estimates we used an apportioning method described in the text.
Human resources	Endemic disease control agents and microscopists	# of agents registered in SIVEP-Malaria * salary of community health workers (based on Law 13,708) [23]	The number of professionals was obtained using the Agent Code from SIVEP-Malaria. We assumed that endemic disease control agents and microscopists allocate all their work time to malaria activities
	Incentives to microscopists	Monetary transfers made by the Ministry of Health to expand the number of microscopists in malaria endemic areas	These incentives occurred only from 2015 to 2017

**Fig 1:**
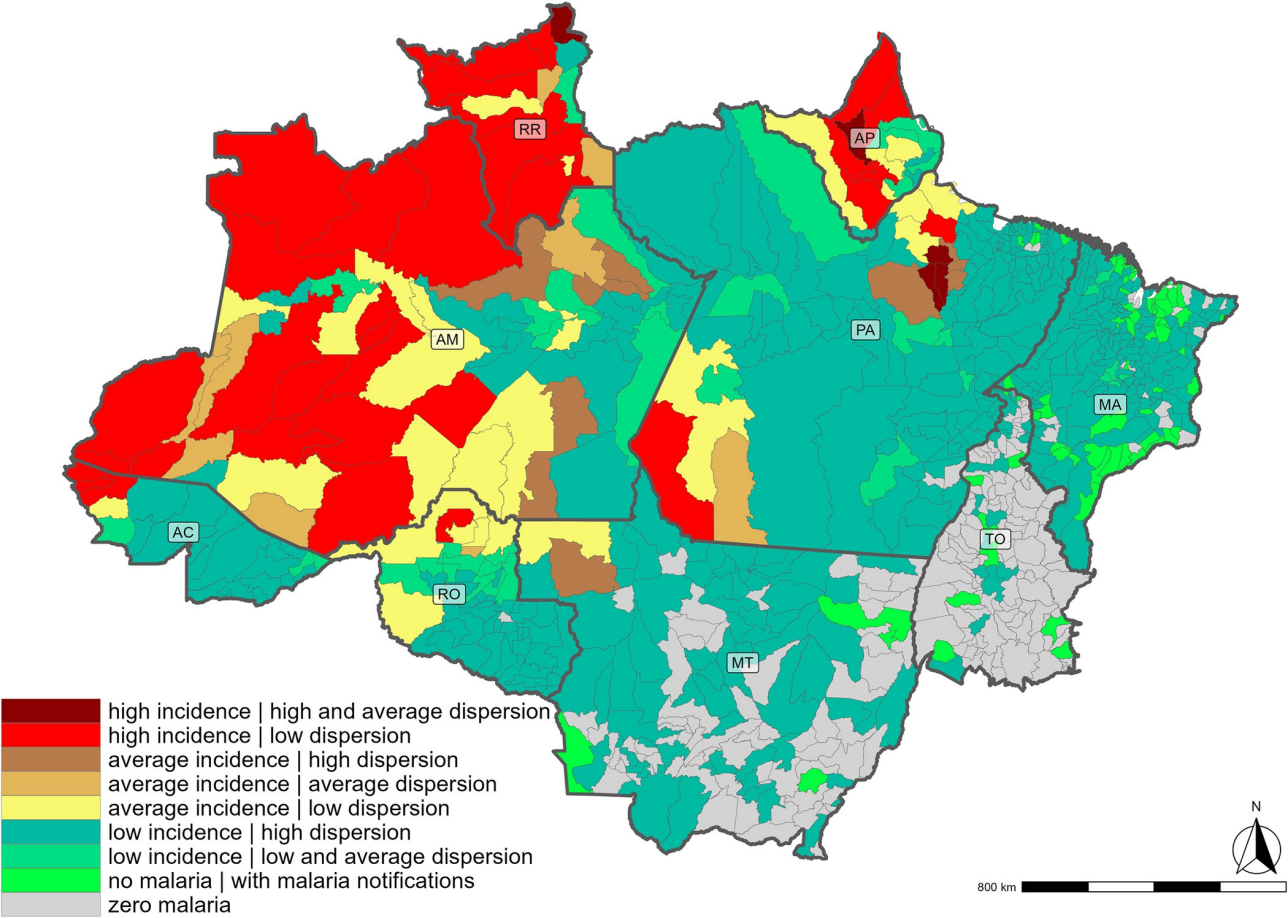
Spatial distribution of groups of municipalities defined according to the apportionment strategy. Note: Maps were created using ggplot package within R environment, version 4.4.0. The base map used is derived from an openly available IBGE shape file source (https://www.ibge.gov.br/geociencias/organizacao-do-territorio/malhas-territoriais.html).

The most difficult cost component to estimate was surveillance. Municipalities have a single budget line for surveillance that does not discriminate by disease or activity. We assume that malaria surveillance costs are proportional to the share of malaria cases among all notifiable infectious diseases. However, the share of malaria cases can fluctuate over the years due to epidemics of any infectious disease. Therefore, we used two variables to group municipalities into categories to facilitate the apportionment of surveillance costs. First, we considered the dispersion (mean divided by the standard deviation) of the share of malaria cases among all notifiable diseases during the study period. Second, we considered the incidence rate, calculated as malaria notifications divided by the total population. S2 Table shows the cut-offs considered for each variable, and the combination of these results in 16 possible groups of municipalities. Due to the small number of observations in some categories, only 8 groups were defined (Fig 1).

Since surveillance expenditures should be incurred even in municipalities with notifications (suspected cases) but no confirmed cases, the category with no malaria (incidence rate = 0) was divided into two subgroups: (i) no malaria cases and no notifications (category “zero malaria” in Fig 1)–here we assumed that municipalities did not allocate any surveillance expenditure to malaria; and (ii) no malaria cases but with notifications (category “no malaria | with malaria notifications” in Fig 1)–here we assumed that municipalities would still allocate some resources to malaria surveillance to provide for testing and control measures, and we hypothesized that 2% of surveillance expenditure would be allocated to malaria even if the average share of malaria cases during the period was zero.

S3 Table shows the number of municipalities, mean, standard deviation, and median share of malaria cases among all notifiable infectious diseases during the study period for each category. The mean percentage was used to determine the apportionment of malaria-related surveillance costs for each group of municipalities. In municipalities with a high or medium incidence of malaria, the percentage of surveillance costs allocated to malaria varied between 63% and 95%.

Given the importance of surveillance in malaria spending and its reliance on an apportionment assumption, we conducted a sensitivity analysis on this component. Specifically, we varied the proportion of surveillance expenditures allocated to malaria by one standard error across the groups defined by the typology.

To summarize the cost of malaria, the following indicators were estimated at the municipal and state levels: total expenditure, per capita expenditure, and expenditure per notification. Another important indicator is the share of malaria expenditures in national public health expenditures. In Brazil, health expenditure accountability is based on the System of Health Accounts methodology, which allows for international comparisons. To calculate this indicator, costs were re-estimated using the reimbursement fees adopted by the System of Health Accounts to estimate health expenditures [24].

Our methodology was discussed and validated with a panel of experts from the National Malaria Control Program (NMCP) at the Ministry of Health. A user-friendly tool was developed to enable health managers at local, state, and national levels to visualize and monitor malaria expenditures. Data manipulation and statistical analysis were performed in R (version 4.0.3) and Stata (version 17). All mapping was done in R (version 4.0.3) using map files of 2020 obtained from the Brazilian Institute of Geography and Statistics (IBGE) using SIRGAS 2000 DATUM.

## Analysis and results

Table 2 shows the malaria expenditure from the perspective of the public health system for the Brazilian Amazon region between 2015 and 2019. The total public expenditure on malaria is approximately PPP-US$ 100 million, reaching PPP-US$ 106.47 in 2019. The results suggest a relationship between the total expenditure and the number of malaria cases. For example, during the 2015–2016 biennium, an increase in expenditure was observed alongside a decrease in malaria cases. Conversely, in the following biennium (2016–2017), a decrease in expenditure was accompanied by an increase in the number of cases. In 2017, when the number of malaria cases peaked, the lowest total health expenditure was recorded. A similar pattern is observed for the number of notifications. Over the analysis period, malaria expenditure account for approximately 0.08% of total expenditure (S4 Table).

**Table 2:** Total malaria expenditure from public health system perspective by cost components, between 2015–2019 (PPP-US$ 2020 million).

Cost componentes	2015		2016		2017		2018		2019	
	US$	%	US$	%	US$	%	US$	%	US$	%
**Illness/treatment**										
Diagnostic tests	2.29	2.41	1.82	1.84	2.11	2.33	2.18	2.17	1.89	1.78
Doctor appointments	0.01	0.01	0.04	0.04	0.06	0.07	0.07	0.07	0.05	0.05
Inpatient care	0.25	0.26	0.24	0.24	0.28	0.31	0.30	0.30	0.28	0.26
Drugs	0.06	0.06	0.10	0.10	0.17	0.19	0.17	0.17	0.15	0.14
**Control and Preventive Actions**										
Insecticide/Bed nets	1.85	1.95	0.54	0.55	2.39	2.64	3.69	3.68	2.78	2.61
Blood screening	0.66	0.70	0.62	0.63	0.57	0.63	0.53	0.53	0.53	0.50
Surveillance	74.02	78.01	82.80	83.84	71.60	79.07	84.27	83.93	89.72	84.27
**Human Resources**										
Agents/Microscopists	14.13	14.89	12.48	12.64	12.43	13.73	9.13	9.09	11.07	10.40
**Total**	94.89	100	98.76	100	90.55	100	100.4	100	106.47	100
Δ% of total expenditures (t+1,t)			4,08		-8,31		10,88		6,05	
# of malaria cases	142,671		128,724		193,874		193,797		156,834	
Δ% of total cases (t+1,t)			-9,78		50,61		-0,04		-19,07	
**# of malaria notifcations**	1,501,501		1,287,104		1,534,852		1,614,047		1,457,099	
**Notifications per 1000 inhab**	54.23		45.96		54.19		56.32		50.26	

S5 Table presents the results of the sensitivity analysis considering a variation of one standard error in the share of malaria surveillance expenditure. Total expenditures in the Amazon region varied from US$91 million to US$98 million in 2015, and from US$102 million to US$110 million in 2019. The variation is less than 10% for all states.

The composition of malaria expenditure across the three domains remains relatively stable over the years. The main cost domain is control and prevention, driven by surveillance expenditure, which alone accounts for 78% of total expenditure in 2015, rising to 84% in 2019. Human resources is the second most important domain, accounting for approximately 9.0% to 15% of the total malaria-related expenditures. Illness/treatment domain accounts for less than 3%, with diagnostic tests as the main cost component (Table 2).

Table 3 shows malaria expenditures per notification and per capita for the Brazilian Amazon as a whole and for its states. Expenditure per notification in the region ranged from PPP-US$59.00 (2017) to PPP-US$77.00 (2016), while per capita expenditure remained relatively stable at around PPP-US$3.50. Notably, Roraima, Amapá, Amazonas, Rondônia, and Acre had the highest per capita expenditures and the lowest expenditures per notification, as these states had the highest incidence rates of malaria notifications per 1000 inhabitants (Fig 1). For example, the notifications per 1000 inhabitants ranged from 182 (2019) to 322 (2015) in Acre and from 187 (2016) to 249 (2019) in Roraima. Tocantins stands out as the state with the highest expenditure per notification over the years followed by Mato Grosso and Maranhão. This high amount is due to the relatively low number of notifications recorded in the state, less than 2 per 1000 inhabitants. It is worth noting that all municipalities located in the these three states are classified as having low incidence or no malaria cases (Fig 1 and S3 Table). Since the malaria epidemic is relatively under control, the per capita expenditure in Tocantins is low compared to other states.

**Table 3:** Malaria expenditure from public health system perspective, between 2015–2019, per notification and per capita (PPP-US$ 2020), by states of the Brazilian Amazon.

Year	Acre	Amapá	Amazonas	Maranhão	Mato Grosso	Pará	Rondônia	Roraima	Tocantins	Amazon Region
**Per notification**										
2015	12.93	92.72	40.81	226.28	216.57	129.24	117.37	79.71	4483.54	63.20
2016	14.04	128.58	58.14	299.36	248.67	117.79	151.08	80.02	2335.86	76.73
2017	15.47	96.44	40.10	247.09	315.28	75.17	138.91	73.18	1021.78	58.99
2018	19.12	116.21	42.93	227.32	395.65	76.98	146.19	60.85	591.32	62.20
2019	25.63	155.5	51.83	291.12	497.93	84.37	125.83	67.01	1979.89	73.07
**Per capita**										
2015	4.16	8.26	7.84	1.10	1.10	2.71	6.68	15.15	1.20	3.43
2016	4.42	9.25	8.45	1.18	1.41	2.22	7.66	14.91	1.20	3.53
2017	4.36	8.07	7.65	1.06	1.25	2.06	6.33	14.00	1.23	3.20
2018	4.99	9.43	8.48	1.18	1.31	2.19	6.68	14.77	1.21	3.50
2019	4.68	10.41	9.23	1.25	1.36	2.20	6.03	16.65	1.22	3.67

Control and preventive activities are the largest cost domain for all states, followed by human resources and illness/treatment. The importance of each domain is closely related to the incidence of malaria. The contribution of illness/treatment and human resources expenses tends to be relatively higher in places where malaria is still a problem. For example, in 2019, illness/treatment accounted for 6.3% of the malaria expenses in Acre, followed by 3.0% in Amazonas and Roraima. The contribution of human resources in these three states is, 11.7%, 15.7%, and 9.5%, respectively. In contrast, in Tocantins and Mato Grosso, these figures are less than 1% (illness/treatment) and 3% (human resources), since the disease is relatively under control. Therefore, the share of control and prevention activities in total malaria expenditure is more pronounced in these places (Table 4). S6 Table shows the data for 2016, 2017 and 2018.

**Table 4:** Distribution of malaria expenses from public healthcare system perspective by domains for each state of the Brazilian Amazon Region, 2015 and 2019.

States	Total (US$ mill)	Illness/ Treatment (%)	Control/ Preventive (%)	Human Resources (%)	Malaria cases
**2015**					
Acre	3.46	13.2	65.2	21.6	27,139
Amapá	6.44	1.5	92.6	5.9	13,657
Amazonas	30.47	4,0	71.1	24.9	75,612
Maranhão	7.58	0.8	87.8	11.4	550
Mato Grosso	3.65	0.8	95.9	3.2	932
Pará	22.38	1.4	87.4	11.2	9,377
Rondônia	11.34	1.7	86.3	12,0	7,386
Roraima	7.77	2.4	70,0	27.6	8,001
Tocantins	1.80	0.1	97.2	2.7	17
Amazon Region	94.89	2.7	80.5	16.7	142,671
**2019**					
Acre	4.13	6.3	82.1	11.7	13,337
Amapá	8.80	1.1	93.7	5.2	10,501
Amazonas	38.25	2.9	81.6	15.5	63,731
Maranhão	8.84	0.5	91.6	7.9	607
Mato Grosso	4.76	0.6	97,0	2.4	1,720
Pará	18.96	1.9	88.4	9.6	32,473
Rondônia	10.72	1.8	92.6	5.7	11,640
Roraima	10.09	3,00	87.5	9.5	22,794
Tocantins	1.93	0.1	97,0	2.8	31
Amazon Region	106.47	2.2	87.3	10.5	156,834

S2 Fig shows the total number of malaria notifications and the spatial distribution of malaria expenditures per capita and per notification by year for all municipalities in the Brazilian Amazon. Expenditures per notification vary significantly between municipalities. In 2019, 77 municipalities had no malaria expenditures, the majority of which (N = 72) are located in Mato Grosso. In contrast, 126 municipalities (dark orange areas) had expenditures per notification higher than PPP-US$863.00. These municipalities are located in Maranhão (50), Pará (38), Tocantins (15), and Mato Grosso (15). These municipalities are adjacent to municipalities without malaria notifications (grey areas). Municipalities with low expenditures (less than PPP-US$67.00) per notification are mainly located in Amazonas (48), followed by Maranhão (29) and Pará (20).

## Conclusion

The aim of this study was to estimate the costs of malaria in Brazil from the perspective of the public health system from 2015 to 2019. To the best of our knowledge, this is the first comprehensive assessment of malaria expenditures in Brazil, with fine spatial and temporal scales, disaggregated by type of cost. We estimate that control and prevention activities account for more than 80% of the malaria-related expenditures. Overall, the cost per malaria notification in the Brazilian Amazon in 2019 was estimated at US$ 73.07 while the cost per capita was US$ 3.67 (both measures as PPP).

Our results highlight the large variation in costs per malaria notification. In 2019, they ranged from US$ 25.63 in Acre to US$ 1,979.89 in Tocantins. This is due to the level of malaria incidence and therefore the variation in whether a suspected case is actually malaria. For example, in 2019, Acre had an API of 15.77% and a the test positivity rate of 8.27%. In contrast, in Tocantins, the API was 0.003%, and the positivity rate was 3.18%. Such variability highlights the challenge of making the economic case for investment in malaria control when transmission is reduced to very low levels [25]. An important potential contribution of this study is the use of information to monitor municipalities in their efforts to eliminate malaria. Tracking notifications and expenditures in municipalities with low incidence rates can help to analyze the minimum expenditures needed to effectively control and ultimately eliminate malaria.

To increase the impact of the cost estimates we developed a user-friendly and interactive digital platform that presents our results disaggregated by state, municipality, year, and cost type. The platform was presented to the NMCP in Brazil. Several improvements were made based on the feedback received by the NCMP team. Future plans for the platform are twofold. First, to work with the NMCP to develop a routine to automatically update the estimates on an annual basis. Second, to disseminate the platform to states and municipalities. With this platform, malaria control program managers and policy makers at all levels of decision-making (municipal, state, and national) will be able to routinely access malaria costs in a timely manner and use these data to set priorities, allocate resources, select control and prevention strategies, and evaluate the cost-effectiveness of interventions.

The period from 2015 to 2019 was chosen because 2019 was the last year with available data before the onset of the pandemic. The COVID-19 pandemic temporarily altered resource allocation and health care delivery, thereby distorting the analysis of expenditures, costs, and utilization. Although 2019 serves as the final year of our analysis, the primary contribution of this paper is the development of a methodology to estimate malaria-related expenditures from the perspective of the public healthcare system using official and publicly available data. This methodology can be easily adapted to reflect changes in treatment and screening protocols. For example, in 2023, the Ministry of Health recommended the use of tafenoquine along with G6PD screening for individuals over 15 year age. Expenditures associated with this new treatment protocol can be easily tracked because SIVEP records drug prescriptions [26, 27].

While we have provided estimates of the financial costs of malaria from a health system perspective, it is likely that the costs from a societal perspective are even higher. These include the costs of school and work absenteeism and presenteeism due to malaria as well as out-of-pocket expenses, to name a few. Empirical evidence has shown that the number of days of work lost per episode of malaria can range from 1.3 days in Brazil [13] to 11 days in India [28]. In addition, the recurrence of malaria episodes can have profound economic consequences for families. Lost workdays can be a major economic burden for families. In Vietnam, lost workdays due to malaria treatment account for 2% of the total annual household income [29].

The strengths of this study include its novelty, use of multiple data sources, disaggregation by cost type, multi-year calculation, disaggregation of costs by municipality and state, and the development of an interactive and user-friendly tool that can assist malaria control managers at different levels of decision making (municipal, state, and federal).

This study has some limitations. Surveillance costs are not available disaggregated by disease. Our assumption that costs are allocated in proportion to disease incidence is the most reasonable given data limitations. However, it is possible that in some municipalities the allocation could be higher or lower depending on competing health priorities and management decisions. Second, the cost estimated for malaria include only federal and municipal expenditures, which are the main public funders. However, state governments may also provide resources, particularly for the organization and planning of surveillance activities. Given the large heterogeneity in the role of these entities in the prevention, control and treatment of malaria in the Brazilian Amazon region, it was not possible to include these expenditures. Third, it was not possible to disaggregate the surveillance and control domain by its inputs, such as fuel, vehicles and equipment. Finally, the estimate did not include the costs associated with the training, recruitment, and retention of health workers within the human resources component. These costs may vary depending on factors such as turnover rates, specific recruitment needs, or training requirements, and therefore may not be easily quantifiable or observable within a systematic framework.

Brazil has set a goal of eliminating malaria by 2035 [30]. The malaria cost estimates presented here contribute to a better negotiation of the financial resources needed by the NMCP and are one of the critical pieces of information for a strategic plan of activities needed to achieve the elimination goal.

## Supporting information

S1 Fig Conceptual framework for malaria expenditure from a public health perspective. (JPG)

S2 Fig Panel (a) Spatial distribution of total malaria notifications, Brazilian Amazon, 2015–2019; Panel (b) Per capita malaria expenditure from the perspective of the public health system between 2015–2019; Panel (c) Malaria expenditures per notification from public healthcare system perspective between 2015–2019 - (PPP-US$ 2020). Note: Maps were created using ggplot package within R environment, version 4.4.0. The base map used is derived from an openly available IBGE shape file source (https://www.ibge.gov.br/geociencias/organizacao-do-territorio/malhas-territoriais.html).
(PNG)

S1 Table Data sources used in the analysis. (DOCX)

S2 Table Categories and corresponding ranges of each criterion used to group municipalities. (DOCX)

S3 Table Mean and median share of malaria cases among all notifiable diseases. (DOCX)

S4 Table Malaria expenditure (PPP-US$ 2020) from the perspective of the public health system and its share in total SUS health expenditure estimated according to SHA accounts, 2015–2019. (DOCX)

S5 Table Results of sensitivity analysis 2015–2019. (DOCX)

S6 Table Composition of malaria expenditure (PPP-US$ 2020) from a public health system perspective and number of malaria cases, by state of the Brazilian Amazon, 2016–2018. (DOCX)
