# Supplementary figures and images for: The economic cost of malaria in Brazil from the perspective of the public health system
Source: PLOS Glob Public Health. 2024 Oct 18;4(10):e0003783. doi: 10.1371/journal.pgph.0003783 (PMC11488710; doi:10.1371/journal.pgph.0003783)

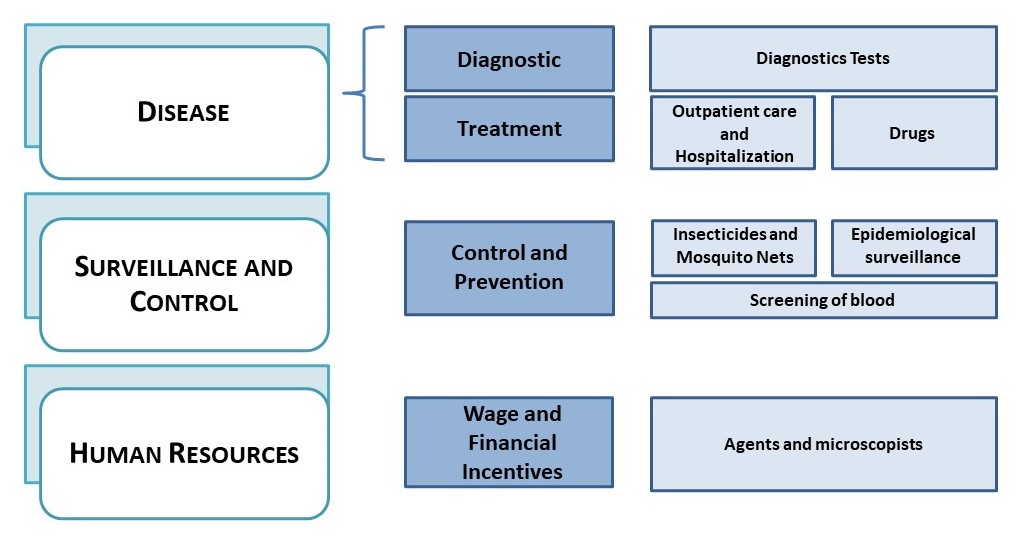

Supplement: S1 Fig — (JPG) [file pgph.0003783.s001.jpg]

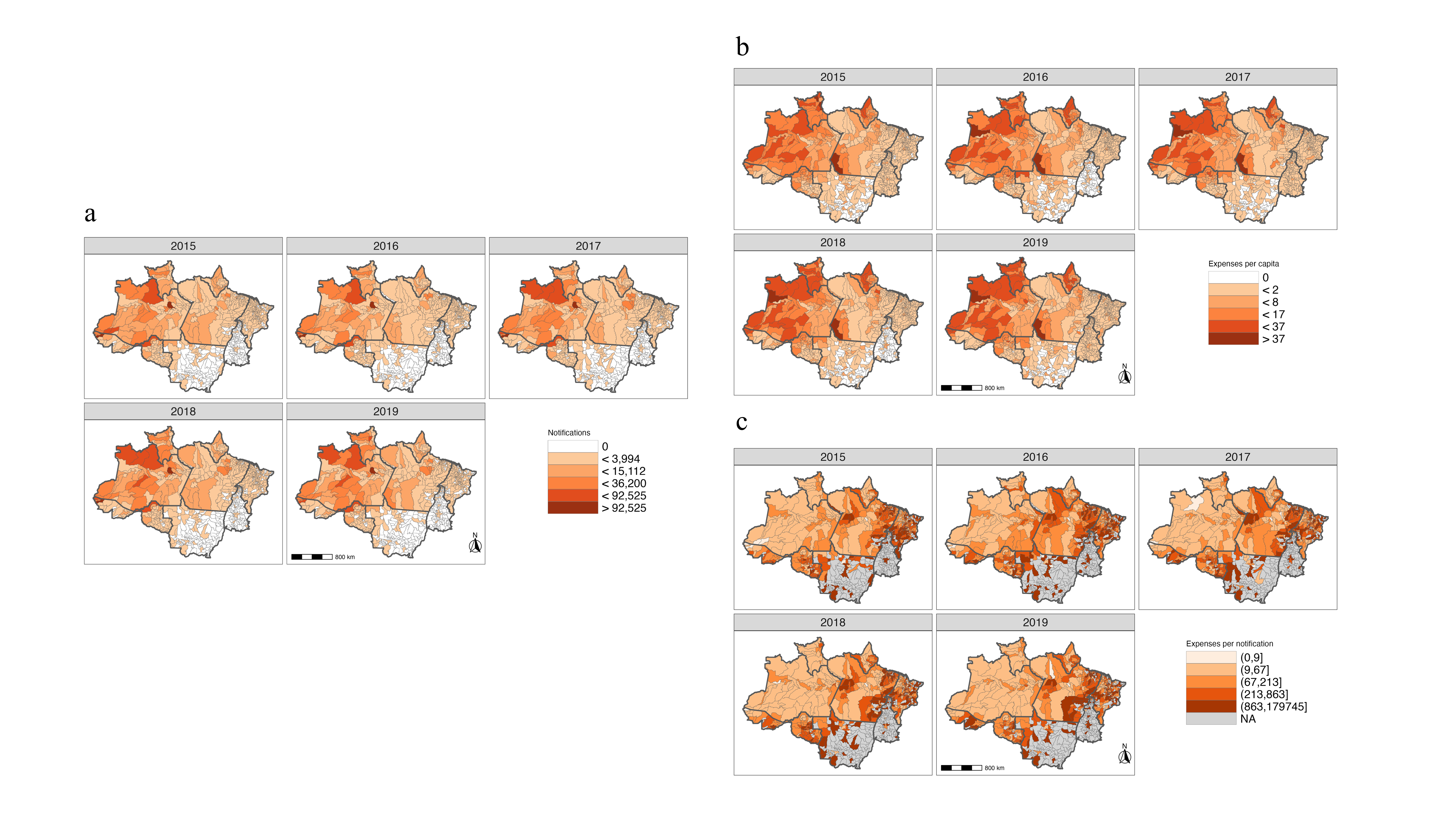

Supplement: S2 Fig — Panel (a) Spatial distribution of total malaria notifications, Brazilian Amazon, 2015–2019; Panel (b) Per capita malaria expenditure from the perspective of the public health system between 2015–2019; Panel (c) Malaria expenditures per notification from public healthcare system perspective between 2015–2019 - (PPP-US$ 2020). Note: Maps were created using ggplot package within R environment, version 4.4.0. The base map used is derived from an openly available IBGE shape file source (https://www.ibge.gov.br/geociencias/organizacao-do-territorio/malhas-territoriais.html). (PNG) [file pgph.0003783.s002.png]
