# Supplementary material for: The economic cost of malaria in Brazil from the perspective of the public health system
Source: PLOS Glob Public Health. 2024 Oct 18;4(10):e0003783. doi: 10.1371/journal.pgph.0003783 (PMC11488710; doi:10.1371/journal.pgph.0003783)
Supplement: S1 Table — (DOCX) [file pgph.0003783.s003.docx]

| **Data source/link** | **Description** | **Spatial Scale** | **Extracted Information** | **Year** |
| --- | --- | --- | --- | --- |
| Institution: Ministry of Health (MoH) | | | | |
| Malaria Epidemiological Surveillance System (SIVEP-Malaria)  Deidentified data provided by the Ministry of Health specifically for the purpose of this research | Includes all suspected and confirmed malaria cases. | Municipality | Number of confirmed malaria cases; suspected cases with a negative test for malaria; endemic disease control agent code; patient information (age, sex, treatment regimen, type of parasite, municipality of residence) | 2015-2019 |
| Hospital Information System (SIH-SUS)  <http://sihd.datasus.gov.br/principal/index.php> | Includes all records of hospital admissions financed by SUS, | Municipality | Number and cost of hospitalizations due to malaria (ICD codes B50, B54, P37.3, or P137.4.), and patient information (local of residence) |  |
| Outpatient Information System (SIA)  <http://sia.datasus.gov.br/principal/index.php> | Includes all records of outpatient procedures financed by SUS | Municipality | Number of tested blood bags |  |
| Notifiable Diseases Information System (SINAN)  <https://portalsinan.saude.gov.br/> | Includes all suspected and confirmed cases of diseases of mandatory notification | Municipality | Number of confirmed notifications of all diseases except malaria |  |
| Strategic Health Supply Information System (SIES)  Deidentified data provided by the Ministry of Health specifically for the purpose of this research | Official web-based system for the analysis, management, control, and allocation of strategic supplies | Brazil and Federal unit | Medicine for malaria treatment | 2015, 2017-2018 |
|  |  |  | Malaria rapid tests | 2015-2016, 2018-2019 |
|  |  |  | Insecticides for indoor residual spraying (IRS) | 2015, 2016, 2017-2019 |
|  |  |  | Insecticides for truck spraying | 2016-2018 |
|  |  |  | Long-lasting insecticide-treated nets (LLINs) | 2018 |
| National Health Fund (FNS)  <https://portalfns.saude.gov.br/> | System for the management of financial resources allocated to SUS | Municipality | Financial incentives to include microscopists in the primary care team | 2015-2017 |
|  |  |  | surveillance | 2015 - 2019 |
| Table of Procedures, Medications, and Orthoses, Prostheses and Special Materials (SIGTAP)  <http://sigtap.datasus.gov.br/tabela-unificada/app/sec/inicio.jsp> | Official value of reimbursement paid by SUS for medical procedures | Brazil | Information on reimbursement values associated with malaria-related procedures and medicines:  Consultation: SIGTAP code 03.01.01007-2  Malaria diagnostic (thick-blood smear): SIGTAP code 02.02.02.045-2  Hospital procedures to treat malaria: SIGTAP code 303010150 | 2015 - 2019 |
| Chamber of Economic Regulation of Pharmaceuticals (CMED)  <https://www.gov.br/anvisa/pt-br/assuntos/medicamentos/cmed> | Interministerial agency responsible for the economic regulation of the drug market | Brazil | Medication prices | 2015 - 2019 |
| Institution: National Treasury Secretariat | | | | |
| Brazilian System of Municipal Accounting (FINBRA)  <https://siconfi.tesouro.gov.br/siconfi/index.jsf> | Consolidates annual municipal accounting and financial information | Municipality | Expenditures on health surveillance | 2015 - 2019 |
| Institution: Brazilian Institute of Geography and Statistics (IBGE) | | | | |
| Population data  [http://tabnet.datasus.gov.br/cgi/deftohtm.exe?ibge/cnv/poptbr.def /](http://tabnet.datasus.gov.br/cgi/deftohtm.exe?ibge/cnv/poptbr.def%20/) | Population estimates produced annually, as determined by law | Municipality | Population estimates by municipality | 2015 - 2019 |
