# Supplementary material for: The economic cost of malaria in Brazil from the perspective of the public health system
Source: PLOS Glob Public Health. 2024 Oct 18;4(10):e0003783. doi: 10.1371/journal.pgph.0003783 (PMC11488710; doi:10.1371/journal.pgph.0003783)
Supplement: S2 Table — (DOCX) [file pgph.0003783.s004.docx]

| **Dispersion of the share of malaria notifications (average/sd)** | | | **Incidence** | |
| --- | --- | --- | --- | --- |
| **Cut-off point** | **Description** | **Cut-off point** | | **Description** |
| 0 | Zero dispersion | 0 | | Zero Incidence |
| < 15 | Low dispersion | < 10 | | Low Incidence |
| 15 - 30 | Intermediate dispersion | 10 - 50 | | Intermediate incidence |
| > 30 | High dispersion | > 50 | | High incidence |
