# Supplementary material for: The economic cost of malaria in Brazil from the perspective of the public health system
Source: PLOS Glob Public Health. 2024 Oct 18;4(10):e0003783. doi: 10.1371/journal.pgph.0003783 (PMC11488710; doi:10.1371/journal.pgph.0003783)
Supplement: S3 Table — (DOCX) [file pgph.0003783.s005.docx]

| **Categories** | **N** | **Average share** | **sd** | **Median share** |
| --- | --- | --- | --- | --- |
| High incidence \| High and intermediate dispersion | 5 | 76.24 | 5.93 | 76.33 |
| High incidence \| low dispersion | 34 | 95.05 | 3.4 | 96.26 |
| Intermediate incidence \| high dispersion | 10 | 63.12 | 11.33 | 64.29 |
| Intermediate incidence \| low dispersion | 27 | 86.84 | 6.47 | 88.29 |
| Intermediate incidence \| intermediate dispersion | 10 | 75.52 | 6.09 | 75.81 |
| Low incidence \| high dispersion | 414 | 11.17 | 14.48 | 4.51 |
| Low incidence \| low and intermediate dispersion | 38 | 55.28 | 22.26 | 62.12 |
| No malaria \| with malaria notifications | 69 | 0* | 0 | 0 |
| Zero malaria | 201 | 0 | 0 | 0 |
| Total | 808 |  |  |  |

*For this group it is assumed that 2% of surveillance expenses is allocated to malaria control and prevention.
