# Supplementary material for: The economic cost of malaria in Brazil from the perspective of the public health system
Source: PLOS Glob Public Health. 2024 Oct 18;4(10):e0003783. doi: 10.1371/journal.pgph.0003783 (PMC11488710; doi:10.1371/journal.pgph.0003783)
Supplement: S4 Table — (DOCX) [file pgph.0003783.s006.docx]

| **Year** | **Malaria expenditures** | | | **Total SUS expenditures (U$ million)*** | **Malaria/SUS (%)** | **Number of malaria cases** |
| --- | --- | --- | --- | --- | --- | --- |
|  | **Total**  **US$ million** | **Per capita**  **(U$)** | **Per notification**  **(U$)** |  |  |  |
| 2015 | 96.52 | 3.49 | 64.28 | 119,039.00 | 0.08 | 142,671 |
| 2016 | 100.57 | 3.59 | 78.14 | 129,319.51 | 0.08 | 128,724 |
| 2017 | 92.71 | 3.27 | 60.40 | 131,289.24 | 0.07 | 193,874 |
| 2018 | 102.93 | 3.59 | 63.77 | 132,873.00 | 0.08 | 193,797 |
| 2019 | 109.15 | 3.77 | 74.91 | 136,894.70 | 0.08 | 156,834 |

*Estimated according to SHA accounts methodology (Brasil, 2022)
