# Supplementary material for: The economic cost of malaria in Brazil from the perspective of the public health system
Source: PLOS Glob Public Health. 2024 Oct 18;4(10):e0003783. doi: 10.1371/journal.pgph.0003783 (PMC11488710; doi:10.1371/journal.pgph.0003783)
Supplement: S5 Table — (DOCX) [file pgph.0003783.s007.docx]

| **Year** | **Amazon Region** | **Acre** | **Amapá** | **Amazonas** | **Maranhão** | **Mato Grosso** | **Pará** | **Rondônia** | **Roraima** | **Tocantins** |
| --- | --- | --- | --- | --- | --- | --- | --- | --- | --- | --- |
| 2015 | [91.37; 98.42] | [3.39; 3.52] | [6.2; 6.69] | [29.45; 31.49] | [7.17; 7.99] | [3.44; 3.86] | [21.28; 23.48] | [11.09; 11.59] | [7.66; 7.89] | [1.69; 1.91] |
| 2016 | [95.02; 102.5] | [3.64; 3.82] | [7.08; 7.65] | [32.16; 34.65] | [7.73; 8.65] | [4.48; 4.99] | [17.68; 19.36] | [12.84; 13.46] | [7.7; 7.99] | [1.71; 1.93] |
| 2017 | [87.37; 93.72] | [3.66; 3.8] | [6.34; 6.78] | [29.7; 31.72] | [7; 7.77] | [4.05; 4.48] | [16.58; 18.1] | [10.75; 11.26] | [7.52; 7.8] | [1.78; 2] |
| 2018 | [96.72; 104.08] | [4.26; 4.42] | [7.57; 8.07] | [33.35; 35.85] | [7.86; 8.8] | [4.26; 4.77] | [17.83; 19.49] | [11.46; 12.03] | [8.36; 8.67] | [1.76; 1.99] |
| 2019 | [102.44; 110.49] | [4.06; 4.21] | [8.53; 9.07] | [36.78; 39.72] | [8.34; 9.33] | [4.49; 5.02] | [18.08; 19.84] | [10.46; 10.98] | [9.9; 10.27] | [1.81; 2.04] |
| Total | 94.89 | 100 | 98.76 | 100 | 90.55 | 100 | 100.4 | 100 | 106.47 | 100 |
