# Supplementary material for: The economic cost of malaria in Brazil from the perspective of the public health system
Source: PLOS Glob Public Health. 2024 Oct 18;4(10):e0003783. doi: 10.1371/journal.pgph.0003783 (PMC11488710; doi:10.1371/journal.pgph.0003783)
Supplement: S6 Table — (DOCX) [file pgph.0003783.s008.docx]

| **States** | **2016** | | | | |
| --- | --- | --- | --- | --- | --- |
|  | **Total**  **(US$ million)** | **Illness/**  **Treatment (%)** | **Control/Preventive actions (%)** | **Human**  **Resources (%)** | **Malaria**  **cases** |
| Acre | 3.7 | 12.4 | 69.4 | 18.2 | 35,209 |
| Amapá | 7.4 | 1.5 | 92.1 | 6.5 | 12,273 |
| Amazonas | 33.5 | 2.7 | 77.0 | 20.3 | 49,150 |
| Maranhão | 8.2 | 0.6 | 91.0 | 8.4 | 766 |
| Mato Grosso | 4.7 | 0.7 | 97.1 | 2.2 | 522 |
| Pará | 18.5 | 1.5 | 86.6 | 11.9 | 14,489 |
| Rondônia | 13.1 | 1.3 | 90.6 | 8.1 | 7,324 |
| Roraima | 7.8 | 2.5 | 89.2 | 8.3 | 8,969 |
| Tocantins | 1.8 | 0.2 | 97.2 | 2.6 | 22 |
| **States** | **2017** | | | | |
|  | **Total**  **(US$ million)** | **Illness/**  **Treatment (%)** | **Control/Preventive actions (%)** | **Human**  **Resources (%)** | **Malaria**  **cases** |
| Acre | 3.7 | 11.6 | 70.1 | 18.4 | 36,640 |
| Amapá | 6.6 | 1.9 | 90.8 | 7.3 | 15,506 |
| Amazonas | 30.7 | 3.9 | 73.7 | 22.4 | 81,356 |
| Maranhão | 7.4 | 0.7 | 89.2 | 10.1 | 954 |
| Mato Grosso | 4.3 | 0.6 | 96.8 | 2.6 | 572 |
| Pará | 17.3 | 2.4 | 85.3 | 12.3 | 36,881 |
| Rondônia | 11 | 1.4 | 90.2 | 8.4 | 7,813 |
| Roraima | 7.7 | 2.9 | 89.4 | 7.7 | 14,082 |
| Tocantins | 1.9 | 0.3 | 96.5 | 3.2 | 70 |
| **States** | **2018** | | | | |
|  | **Total**  **(US$ million)** | **Illness/**  **Treatment (%)** | **Control/Preventive actions (%)** | **Human**  **Resources (%)** | **Malaria**  **cases** |
| Acre | 4.3 | 8.8 | 80.1 | 11.2 | 26,306 |
| Amapá | 7.8 | 1.5 | 93.5 | 9.0 | 15,246 |
| Amazonas | 34.6 | 3.6 | 82.1 | 14.3 | 71,786 |
| Maranhão | 8.3 | 0.7 | 91.2 | 8.1 | 924 |
| Mato Grosso | 4.5 | 0.5 | 97.7 | 1.7 | 860 |
| Pará | 18.7 | 2.3 | 89.7 | 7.9 | 45,832 |
| Rondônia | 11.7 | 1.4 | 94.3 | 4.3 | 9,450 |
| Roraima | 8.5 | 3.5 | 89.1 | 7.4 | 23,369 |
| Tocantins | 1.9 | 0.5 | 96.1 | 3.4 | 24 |
